# Ancient amino acids from fossil feathers in amber

**DOI:** 10.1038/s41598-019-42938-9

**Published:** 2019-04-23

**Authors:** Victoria E. McCoy, Sarah E. Gabbott, Kirsty Penkman, Matthew J. Collins, Samantha Presslee, John Holt, Harrison Grossman, Bo Wang, Monica M. Solórzano Kraemer, Xavier Delclòs, Enrique Peñalver

**Affiliations:** 1 Steinmann-Institut für Geologie, Mineralogie und Paläontologie, Universität Bonn, Nussallee 8, 53115 Bonn, Germany; 2 School of Geography, Geology, and the Environment, University of Leicester, University Road, Leicester, LE1 7RH UK; 3 Department of Chemistry, University of York, Heslington, York, YO10 5DD UK; 4 BioArCh, Department of Archaeology, University of York, York, YO10 5DD UK; 5 Natural History Museum of Denmark, University of Copenhagen, Østervoldgade 5-7, DK-1350 Copenhagen K, Denmark; 6 Space Research Centre, Department of Physics and Astronomy, University of Leicester, University Road, Leicester, LE1 7RH UK; 7 State Key Laboratory of Palaeobiology and Stratigraphy, Nanjing Institute of Geology and Palaeontology and Center for Excellence in Life and Paleoenvironment, Chinese Academy of Sciences, 39 East Beijing Road, Nanjing, 210008 China; 8 Senckenberg Forschungsinstitut und Naturmuseum, Paläontologie und Historische Geologie, Senckenberganlage 25, 60325 Frankfurt am Main, Germany; 9 Department Dinàmica de la Terra i de l’Oceà, Facultat de Ciències de la Terra, and Institut de Recerca da la Biodiversitat (IRBio), Universitat de Barcelona, 08028 Barcelona, Spain; 10 Instituto Geológico y Minero de España (Museo Geominero), C/Cirilo Amorós, 42–46004 Valencia, Spain

**Keywords:** Palaeontology, Proteomics

## Abstract

Ancient protein analysis is a rapidly developing field of research. Proteins ranging in age from the Quaternary to Jurassic are being used to answer questions about phylogeny, evolution, and extinction. However, these analyses are sometimes contentious, and focus primarily on large vertebrates in sedimentary fossilisation environments; there are few studies of protein preservation in fossils in amber. Here we show exceptionally slow racemisation rates during thermal degradation experiments of resin enclosed feathers, relative to previous thermal degradation experiments of ostrich eggshell, coral skeleton, and limpet shell. We also recover amino acids from two specimens of fossil feathers in amber. The amino acid compositions are broadly similar to those of degraded feathers, but concentrations are very low, suggesting that much of the original protein has been degraded and lost. High levels of racemisation in more apolar, slowly racemising amino acids suggest that some of the amino acids were ancient and therefore original. Our findings indicate that the unique fossilisation environment inside amber shows potential for the recovery of ancient amino acids and proteins.

## Introduction

Biomolecules such as proteins have great potential to provide new evidence for investigating ancient fossil organisms^1,2^. Previous studies of ancient proteins have focused primarily on hard tissue remains (e.g. bone and shell) in sedimentary rocks. However, a focus on biomineralised fossils recovered from sediments limits the taxonomic coverage of such analyses^3–11^; large vertebrates are particularly overrepresented. The recovery of proteins from fossils in amber, which are primarily small, terrestrial soft-bodied organisms not found in sediments^12,13^, would provide an important biomolecular archive from extinct taxa that cannot be matched by the sedimentary fossil record.

Fossil inclusions in amber are characterised by exceptional morphological preservation of soft tissues, which suggest the possibility of similarly exceptional protein preservation^13^; this is supported by two previous investigations of fossils in amber based on levels of amino acid racemisation^14,15^. Amino acids primarily racemise (convert from the left-handed to right-handed chiral forms and vice versa) at N-terminal positions, most racemising very slowly within proteins and relatively more rapidly as free amino acids. Therefore the degree of racemisation in a closed system correlates with the degree to which the protein has degraded^3^. Low levels of racemisation as a result of limited peptide bond hydrolysis may imply that peptides are preserved^3^, or indicate some modern protein contamination. Liquid chromatography analysis of insects in resin, copal, and amber samples, ranging in age from 100 years old to 130 million years old, reported ancient amino acids with very low levels of racemisation^14^. Smejkal, *et al*.^15^ reported peptides from plant inclusions in Dominican amber (~15 Ma), and identified them as originating from ancient fungal proteins. Bada, *et al*.^14^ suggested that amber may provide the ideal environment for protein preservation owing to the dehydrating effects of the resin matrix inhibiting protein hydrolysis.

**Figure 1:**
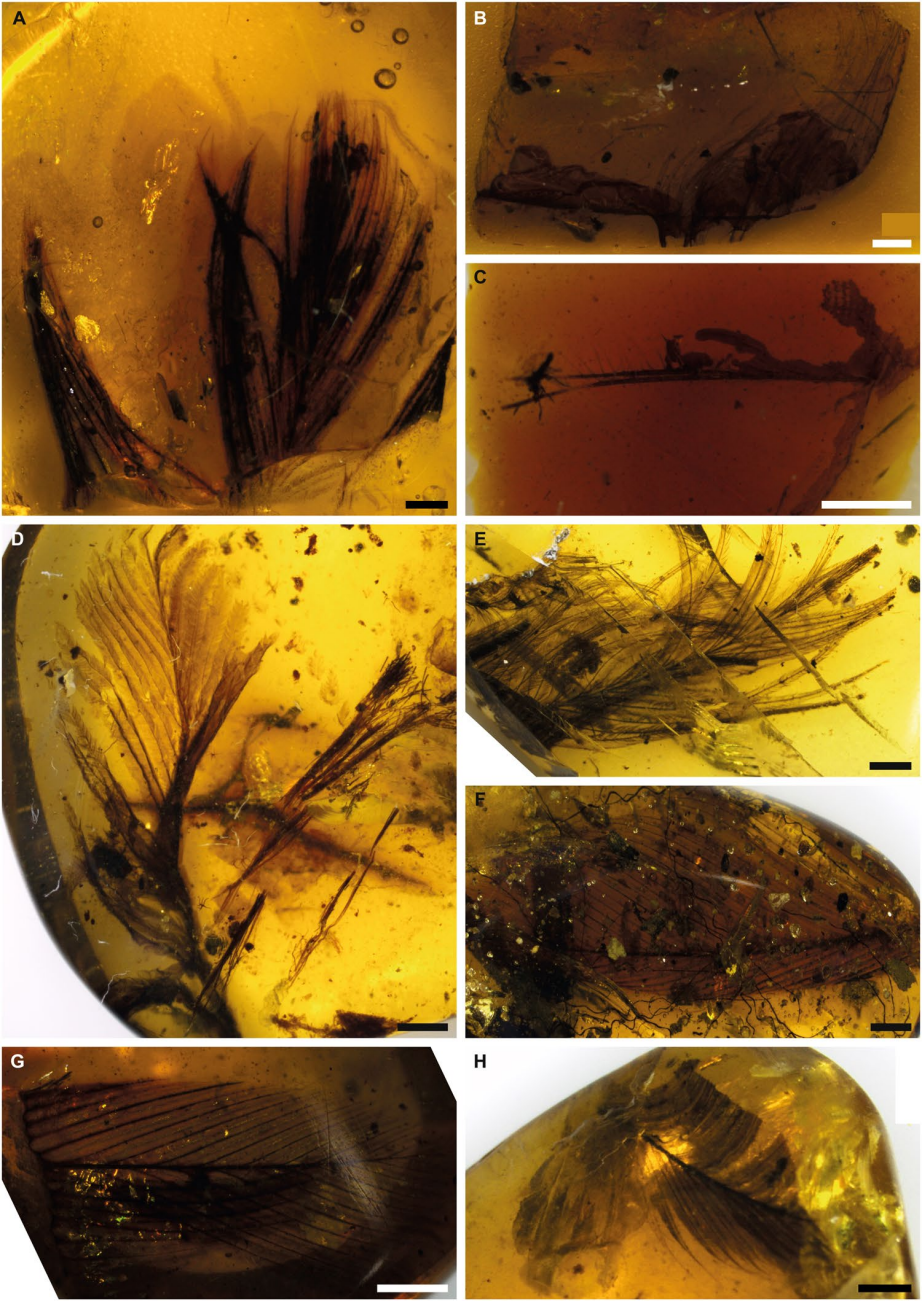
Specimens of feathers in amber used in this study. Scale bars = 1 mm. (**A**) Baltic amber, Smf Be 370, yielded amino acids. (**B**) Spanish amber, CES 457. (**C**) Spanish amber, CES 426. (**D**) Burmese amber, specimen 1. (**E**) Burmese amber, specimen 2, yielded amino acids. (**F**) Burmese amber, specimen 3. (**G**) Burmese amber, specimen 4. (**H**) Burmese amber, specimen 5. The sixth specimen of Burmese amber was not photographed before destructive sampling.

In this study we provide new data from experimental protein degradation and undertake the first analyses of racemisation in ancient feathers in amber (Fig. 1). We focus on feathers because they are composed almost entirely of the protein beta-keratin, and the evolution of the various keratin proteins that comprise feathers (and other integumentary structures in vertebrates), have been widely explored and debated^16,17^. Thus, identification of protein sequences from fossil feathers, combined with their morphological investigation, would allow important functional and evolutionary information to be determined over long timescales. Moreover, the preservation potential of keratin in different environments has been the subject of many recent papers^18–26^. Generally speaking, keratin is thought to have a high preservation potential in sedimentary environments, as suggested by the results of burial experiments and analyses of a number of fossil feathers^18,19,22,24–27^. However, experiments focusing on diagenetic alteration report rapid degradation of keratin under increased pressure and temperature^20,23^. Finally, some bacteria are more effective than others at degrading keratin^21^. Based on these apparently contradictory results, the fossilisation of keratin is most likely complex and strongly influenced by environmental chemistry; investigating the preservation of feathers in the specific chemical environment in amber will help us further untangle this complex process. In addition to the analysis of fossil feathers, we also conducted thermal degradation experiments on chicken feathers in resin (the precursor to amber) from the modern Araucariacean gymnosperm *Wollemia nobilis*. This provides crucial additional evidence for interpreting the fossil results by taking into account the environmental framework of protein decay and preservation in resin.

## Results and Discussion

**Figure 2:**
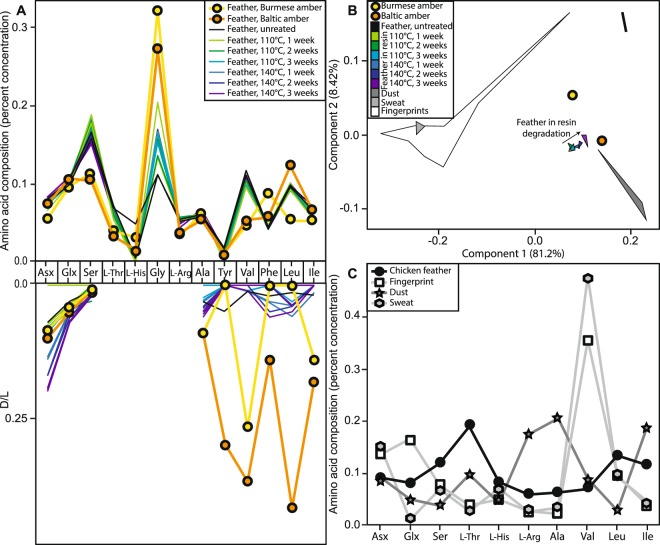
Amino acid analyses. (**A**) Amino acid compositions and D/L values for the taphonomic experiments and fossil analyses. (**B**,**C**) Comparisons of the amino acid compositions of taphonomic experiments and fossil analyses (this paper) to dust^49^, human sweat^50,51^, and human fingerprints/fingertips^33,34,52^, using only the amino acids Asx, Glx, Ser, Thr, His, Arg, Ala, Val, Leu, Ile. These 10 amino acids were the only ones for which previously-published data was provided for common amino acid contaminants; the compositions from our experimental and analytical samples were recalculated using only these ten amino acids. (**B**) Principal component analysis (PCA). (**C**) Amino acid compositions.

**Figure 3:**
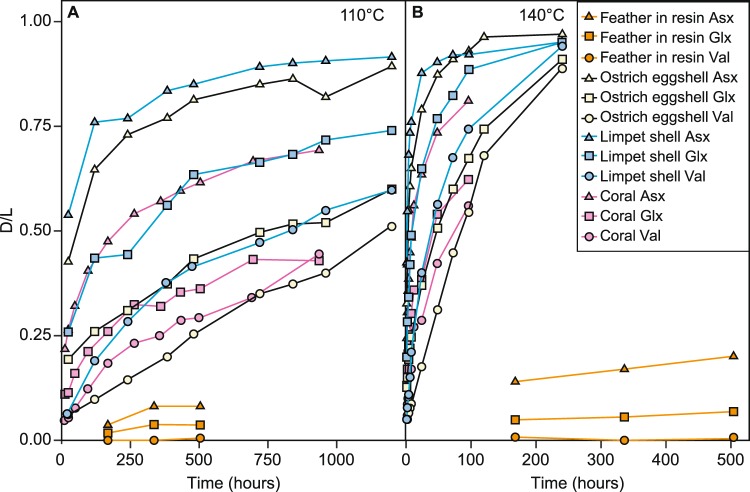
Mean D/L values of Asx, Glx, and Val during thermal maturation experiments of modern samples, including *Gallus g. domesticus* feathers in resin (this paper), ostrich eggshell^28^, limpet shell^30^, and coral^29^. (**A**) 110 °C. (**B**) 140 °C. Note that the other experiments (ostrich eggshell, limpet shell, and coral) included environmental water, whereas the experiments reported in this paper (feathers in resin) did not include any environmental water.

Using chiral amino acid analysis by reverse-phase high pressure liquid chromatography (RP-HPLC), we assessed the rate of amino acid degradation during thermal degradation of modern chicken feathers heated dry in modern *Wollemia nobilis* resin. Despite a slew of recent papers investigating protein fossilisation^3,4,28,29^ protein degradation is still not fully understood, and this is especially true in the unusual chemical environment found in amber. The experiments therefore give us a framework to understand the patterns of amino acid racemisation, and changes in amino acid composition, during fossilisation in amber. These experimentally aged feathers in resin showed very low levels of protein degradation, with D/L < 0.5 for all amino acids in all samples, even after 504 hours at 140 °C (Fig. 2, Table S3), comparable to or slightly lower than the levels of protein degradation seen in feathers thermally degraded in air (Fig. S1, Tables S4 and S5). Significantly, this is considerably slower than rates of protein degradation in comparable experiments on other sample types which contained closed system protein, including ostrich eggshell^28^, coral^29^, and mollusc shells^30^ (Fig. 3). The modern chicken feathers in resin also showed a decrease in concentration (Table S2) and a continuous increase in racemisation of amino acids with increased heating time and increased temperature (Fig. 2).

We also investigated nine fossil specimens (ranging from 44 Ma to 105 Ma in age, Supplemental Table S1, Fig. 1). In seven cases, we could not detect amino acids, suggesting that in these samples all the protein had degraded. Two samples, one each from Eocene (~44 Ma) Baltic amber (Fig. 1A)^31^ and Cenomanian (~99 Ma) Burmese amber (Fig. 1E)^32^, had detectable amino acids; of these two samples, we recovered lower concentrations of amino acids from the older Burmese amber feather than from the younger Baltic amber feather (Supplemental Table S2). The primary goal in analysing these amino acids is to rigorously test the null hypothesis that they represent contamination rather than ancient, endogenous, amino acids.

The sampling and analytical methods were carefully developed to prevent introducing modern contamination during our handling of the specimens. The lack of detectable amino acids in seven of our nine samples suggests that this was successful; if modern contamination was introduced during sampling, all samples would likely have been affected.

Although we limited the introduction of contamination during our sampling, modern contamination could also have been introduced by others between the time of collection and analysis. In this case, we would expect the composition of the amino acids to be more similar to a contaminant than to feathers. Human keratin would be the most likely contaminant; previous studies have indicated that contamination of fossils with human keratin primarily occurs through direct skin contact (e.g. the fingerprint/fingertip amino acids) or through keratin dust as part of laboratory dust^33,34^. The proteins present in the fingerprints/fingertips and sweat are primarily human keratin^33,35^; these both provide a direct test of human keratin contamination. Laboratory dust is a mixture of human keratin and proteins from other sources^33,34^; including multiple samples of laboratory dust provides a range of comparative mixtures. Other possible contaminants can be introduced during cleaning fossils, keratin from animal hair brushes used to clean fossils or protein-containing glue used to repair fossils; however any brushes would have only made contact with the outer surface of the amber, which we removed prior to analysis, and these samples were not glued. The amino acid composition of the feathers (both fresh and experimentally degraded in resin) does not overlap with any of the contaminant samples including human keratin (Fig. 2), indicating that contamination can be distinguished from feather amino acids. Both fossil amino acid samples are more similar to modern chicken feathers degraded in resin than to dust, fingerprints/fingertips, or sweat, a few of the most likely modern contaminants (Fig. 2), suggesting these amino acids were indeed from the enclosed fossil feather. The Baltic amber sample specifically falls exactly along the line of experimental feather degradation in resin (Fig. 2), indicating that the composition is exactly what we would expect for a feather in resin that is slightly more degraded than in our experiments. The Burmese amber sample does not so clearly match feathers degraded in resin, but it falls between fresh feathers and feathers degraded in resin, and is not similar to any contaminant (Fig. 2).

More evidence that at least some of this signal was from ancient amino acids came from the pattern of amino acid racemisation, which can indicate whether amino acids are modern or ancient. The degree of racemisation in some hydrophobic amino acids, such as Tyr, Val, Phe, Leu, Ile (particularly in the Baltic amber sample), is much higher than would be expected, or indeed than would be possible, for modern contamination (Supplemental Table S3, Fig. 2). The fossils have undergone much more extensive decay than the experimental samples, and should therefore have higher D/L values. This is the case for the high D/L values of the hydrophobic amino acids (e.g. Tyr, Val, Phe, Leu, Ile) recovered from the two fossil feathers (Table S3, Fig. 2), which strongly suggests they are (at least in large part) ancient. In contrast, the D/L values of the faster racemising amino acids (e.g. Asx and Ser) are comparatively low, inconsistent with the data from apolar amino acids, and from the experiments; we would typically expect fossils to exhibit high degrees of racemisation of these amino acids, and to preferentially lose polar amino acids as well as the most unstable amino acids^28,30,36,37^. The Burmese amber sample shows less racemisation than the Baltic amber sample (Fig. 2, Table S3), which is unexpected given that Burmese amber (~99 Ma) is approximately twice as old as Baltic amber (~44 Ma). This could be explained by some contamination, or by the degradation of the free amino acids themselves, which would preferentially remove right-handed amino acids from the samples. Significant contamination, however, is unlikely due to the strong feather signal in the amino acid composition. Moreover, other researchers have found that keratin proteins preserve even into the Jurassic^27^, suggesting that Cretaceous keratin preservation is not at all unexpected or unreasonable.

The low concentrations of amino acids in all amber samples imply that amber-entombed feathers have undergone significant diagenesis. However the variable levels of recovered amino acids further support the idea that not all fossils are equal; that two of nine samples included detectable levels is encouraging. CT and synchrotron images of amber-entombed insects sometimes demonstrate loss of the internal tissue^38^, suggesting that decay proceeds rapidly following entombment when there is still sufficient entrapped water to enable decay.

As amino acids from the Burmese amber feather (~99 Ma) are less racemised than the amino acids from the younger Baltic amber sample (~44 Ma), this suggests that the methods of entrapment result in multiple independent diagenetic histories. A number of variables have the potential to affect protein degradation rate. For example, the distinct resin chemistries of different ambers are indicative of preservation fidelity; some resins are characterised by almost perfect preservation, others by poor preservation^38^. This bias will act on proteins alongside other tissue compositions. In particular, fruit flies decay very quickly in *Pinus* resin (Pinaceae) and very slowly in *Wollemia* resin (Araucariaceae)^39^. Baltic and Burmese amber are chemically distinct^31,40–42^. Burmese amber is produced by an Araucareacean, phylogenetically close to *Wollemia*^42^, and although the botanic source of Baltic amber is disputed^31^, a number of authors have noted it likely belongs to the Pinaceae family^31,43^. It is possible that the decreased racemisation of the older Burmese amber, compared with the Baltic amber, could reflect a reduced decay rate due to variations in the amber chemical microenvironment and therefore its antimicrobial effects^44^.

In conclusion, the combination of careful sampling procedures, amino acid composition data, and amino acid racemisation data suggests that we found potentially ancient, endogenous amino acids from fossil feathers in amber. The amino acids we recovered were similar to those previously identified from insects in Baltic amber^14^: both showed low amino acid concentration, significant degrees of racemisation, and the presence of some reactive, polar amino acids. Furthermore, the amino acid composition of the fossils was similar to the amino acid composition of chicken feathers thermally aged in modern resin. However, the D/L values, which were higher in the younger fossil feather than in the older fossil feather, and the high levels of some polar, reactive amino acid in the fossils are not exactly what we would expect based on the experiments. The process of protein fossilisation and degradation in amber requires further study to determine if these discrepancies are due to partial amino acid contamination, or whether the experiments (3 weeks of thermal aging) do not encompass all possible pathways of chemical change and degradation in amber.

## Methods

### Artificial aging experiments

Our artificial aging experiment on modern feathers used freshly plucked *Gallus gallus domesticus* (domestic chicken) feathers which were embedded in freshly extruded *Wollemia nobilis* resin. *Wollemia nobilis* trees were purchased from wollemipine.co.uk, and liquid resin was collected from these trees. *Wollemia nobilis* is an Araucariacean, a family of trees known to produce extensive amber, copal, and resin deposits from the Cretaceous to the Recent, including both Burmese and Spanish amber^40,42,44^; although there are some chemical similarities between Araucariaceae resin and Baltic amber, the botanic origin of this amber is still widely disputed^30,43,45^. A small amount of liquid resin was dripped onto a glass slide, and chicken feather fragments were then mixed into the resin. The mixture of resin and feather was then coated with more resin, to ensure that the feather was not exposed on the surface of the resin. Heating at 110 °C or 140 °C is commonly used as means of accelerating degradation in order to replicate over laboratory timescales the changes that occur during fossilisation^28–30^. Each experiment was thermally aged in a convection oven (Binder) for 1, 2 or 3 weeks at 110 °C or 140 °C. At each sampling interval, feathers in resin were analysed for AAR allowing us to track the rates and patterns of feather protein (keratin) degradation in resin. We did not include environmental water in these experiments, even though that is a common component of such experiments^28–30^, because: (1) in the presence of water, the resin melted at both 110 °C and 140 °C so it no longer protected the feather, and (2) in the previously published closed system experiments mentioned above, the environmental water does not penetrate into the intra-crystalline fraction of amino acids analysed.

### Fossil preparation

Fossil feathers in amber (Fig. 1, Supplemental Table S1) were obtained from various sites: two specimens from Cretaceous (Albian) Spanish amber (~105 Ma)^46,47^, CES 426 and CES 457 from the laboratory of the El Soplao Cave, Celis, Cantabria (Spain) encompassing the Institutional Collection from the El Soplao outcrop; six un-numbered specimens from Cretaceous (earliest Cenomanian) Burmese amber (~99 Ma)^32,40^ from the Nanjing Institute of Geology and Paleontology in China (numbers were not assigned because they were not holotypes or paratypes); and one specimen from Eocene (Lutetian) Baltic amber (~44 Ma)^31^, SMF Be 370 from Senckenberg Research Institute and Natural History Museum, Frankfurt. Cross contamination was minimised and sampling conducted in an aseptically managed, class 1000 cleanroom at the University of Leicester^48^ (typical monitoring of the working environment is better than class 100). All tools that made contact with the amber were cleaned either with a heat treatment (6 hours in a dry furnace at 400 °C) or, for tools that could not survive the heat treatment, a chemical treatment (12% NaOCl (VWR), HPLC-grade water (VWR) and HPLC-grade methanol (VWR)). The surface of each piece of amber, along with any surficial protein contamination, was removed with an aluminum oxide Dremel grinding stone, and then a piece of the amber and feather was removed from the specimen using a synthetic diamond Dremel cutting wheel. Each cut piece was pulverised inside a sterile aluminum foil pocket with a hammer to expose the feather at the surface. Further grinding with an agate mortar and pestle was used to separate the feather from the amber as much as possible, and the fragments of feathers were then transferred to a sterile glass vial for amino acid racemisation (AAR) analysis, to determine if ancient amino acids were present, and if so whether these are still bound into proteins or peptides. An initial, small sample of feather was prepared from each specimen and comprised the target feather and small amounts of amber. These samples were tested for amino acid yield (see below). Samples that yielded amino acids above the baseline were selected for resampling such that larger pieces of feather were extracted, with care taken to exclude amber from these samples. All amino acid composition results were compared to previously published data on common contaminants including dust^49^, human sweat^50,51^, and human fingerprints/fingertips^33,34,52^. The previously published data on common contaminants included only ten amino acids (rather than the thirteen we analysed) and so, in order to compare our results directly to these data, we recalculated our amino acid composition results using only these ten amino acids.

### Amino acid racemisation

Each sample was placed in a sterile glass microvial; 100 μL 7 M HCl (Aristar) was added, the vial was flushed with nitrogen and oven- heated to 110 °C for 24 hours; this is likely to release the maximum concentration of amino acids whilst inducing minimum racemisation^53^. Samples were dried in a centrifugal evaporator. For analysis by reverse-phase HPLC (Agilent 1100) the samples were rehydrated with 50 µL rehydration fluid per mg of original sample. The rehydration fluid (0.01 M HCl, 1.5 mM sodium azide) contains a non-protein amino acid L-homo-arginine at a concentration of 0.01 mM that elutes with baseline separation approximately 50 minutes into the run time. This was used as an internal standard to quantify the concentrations of amino acids in the sample. After rehydration, the samples were spun in a centrifugal evaporator to remove any solid amber debris and the supernatant pipetted off for analysis. The solution was transferred to a sterile autosampler vial with a tapered insert. The amino acid compositions of the samples were analysed by RP-HPLC using fluorescence detection following a modified method of Kaufman and Manley^52^. 2 μL of sample was injected and mixed online with 2.2 μL of derivatising reagent (260 mM N-Iso-L-butyryl L-cysteine (IBLC), 170 mM o-phthaldialdehyde (OPA) in 1 M potassium borate buffer, adjusted to pH 10.4 with potassium hydroxide pellets). The amino acids were separated on a C18 HyperSil BDS column (5 × 250 mm) at 25 °C using a gradient elution of 3 solvents: sodium acetate buffer (solvent A: 23 mM sodium acetate tri-hydrate, 1.5 mM sodium azide, 1.3 μM EDTA, adjusted to pH 6.00 ± 0.01 with 10% acetic acid and sodium hydroxide), methanol (solvent C) and acetonitrile (solvent D). Initially 95% A and 5% C is used at a flow rate of 0.56 mL/min, grading to 50% C and 2% D after 95 minutes. Prior to the injection of the next sample, the column was flushed with 95% C and D for 15 minutes, followed by equilibration of 95% A and 5% C for 5 minutes. The fluorescence detector uses a xenon-arc flash lamp at a frequency of 55 Hz, with a 280 nm cut-off filter and an excitation wavelength of 230 nm and emission wavelength of 445 nm. The L and D isomers of 12 amino acids can be separated, and standards and blanks are routinely analysed. During preparative hydrolysis both asparagine and glutamine undergo rapid irreversible deamination to aspartic acid and glutamic acid respectively^54^. It is therefore not possible to distinguish between the acidic amino acids and their derivatives and they are reported together as Asx and Glx respectively.

## Supplementary information

Supplementary Information for Ancient amino acids from fossil feathers in amber

Supplemental table S2

Supplemental table S3

Supplemental table S4

Supplemental table S5

## Data Availability

The authors declare that all data supporting the findings of this study are available within the paper and its supplement.

## References

[1] Cappellini E, Collins MJ, Gilbert MTP (2014). Unlocking ancient protein palimpsests. Science.

[2] Cleland TP, Schroeter ER (2018). A comparison of common mass spectrometry approaches for paleoproteomics. Journal of Proteome Research.

[3] Demarchi B (2016). Protein sequences bound to mineral surfaces persist into deep time. Elife.

[4] Rybczynski N (2013). Mid-Pliocene warm-period deposits in the High Arctic yield insight into camel evolution. Nat. Commun..

[5] Cleland TP (2015). Mass spectrometry and antibody-based characterization of blood vessels from *Brachylophosaurus canadensis*. J. Proteome Res..

[6] Welker F (2015). Ancient proteins resolve the evolutionary history of Darwin’s South American ungulates. Nature.

[7] Cappellini E (2012). Proteomic analysis of a Pleistocene mammoth femur reveals more than one hundred ancient bone proteins. J. Proteome Res..

[8] Schweitzer MH, Zheng W, Cleland TP, Bern M (2013). Molecular analyses of dinosaur osteocytes support the presence of endogenous molecules. Bone.

[9] Buckley M, Warwood S, van Dongen B, Kitchener AC, Manning PL (2017). A fossil protein chimera; difficulties in discriminating dinosaur peptide sequences from modern cross-contamination. Proc. Biol. Sci..

[10] Wadsworth C, Buckley M (2014). Proteome degradation in fossils: investigating the longevity of protein survival in ancient bone. Rapid Commun. Mass Spectrom..

[11] Schroeter ER, Cleland TP (2016). Glutamine deamidation: an indicator of antiquity, or preservational quality?. Rapid Commun. Mass Spectrom..

[12] Martínez-Delclòs X, Briggs DEG, Peñalver E (2004). Taphonomy of insects in carbonates and amber. Palaeogeogr. Palaeoclimatol. Palaeoecol..

[13] Labandeira CCA (2014). Reading and writing of the fossil record: Preservational pathways to exceptional fossilization. Paleontological Society Papers.

[14] Bada JL, Wang XS, Poinar HN, Paabo S, Poinar GO (1994). Amino acid racemization in amber-entombed insects: implications for DNA preservation. Geochim. Cosmochim. Acta.

[15] Smejkal, G. B., Poinar, G. O., Righetti, P. G. & Chu, F. Revisiting Jurassic Park: The isolation of proteins from amber encapsulated organisms millions of years old. In *Sample Preparation in Biological Mass Spectrometry* 925–938 (Springer, Dordrecht, 2011).

[16] Fraser RDB, Parry DAD (2014). Amino acid sequence homologies in the hard keratins of birds and reptiles, and their implications for molecular structure and physical properties. J. Struct. Biol..

[17] Prum RO, Brush AH (2014). Which came first, the feather or the bird?. Sci. Am..

[18] Schweitzer MH, Zheng W, Moyer AE, Sjövall P, Lindgren J (2018). Preservation potential of keratin in deep time. PLoS One.

[19] Schweitzer MH (1999). Keratin immunoreactivity in the Late Cretaceous bird *Rahonavis ostromi*. J. Vert. Paleontol..

[20] Saitta ET, Rogers CS, Brooker RA, Vinther J (2017). Experimental taphonomy of keratin: A structural analysis of early taphonomic changes. Palaios.

[21] Ichida JM (2001). Bacterial inoculum enhances keratin degradation and biofilm formation in poultry compost. J. Microbiol. Methods.

[22] Schweitzer MH (1999). Beta-keratin specific immunological reactivity in feather-like structures of the Cretaceous Alvarezsaurid, *Shuvuuia deserti*. J. Exp. Zool..

[23] Saitta ET (2017). Low fossilization potential of keratin protein revealed by experimental taphonomy. Palaeontology.

[24] Moyer AE, Zheng W, Schweitzer MH (2016). Keratin durability has implications for the fossil record: Results from a 10 year feather degradation experiment. PLoS One.

[25] Pan Y (2016). Molecular evidence of keratin and melanosomes in feathers of the Early Cretaceous bird *Eoconfuciusornis*. Proc. Natl. Acad. Sci. USA.

[26] Schweitzer MH (2014). A role for iron and oxygen chemistry in preserving soft tissues, cells and molecules from deep time. Proc. Biol. Sci..

[27] Pan, Y. *et al*. The molecular evolution of feathers with direct evidence from fossils. *Proc. Natl. Acad. Sci. USA* 201815703 (2019). 10.1073/pnas.1815703116 PMC6386655 30692253

[28] Crisp M (2013). Isolation of the intra-crystalline proteins and kinetic studies in *Struthio camelus* (ostrich) eggshell for amino acid geochronology. Quat. Geochronol..

[29] Tomiak PJ (2013). Testing the limitations of artificial protein degradation kinetics using known-age massive *Porites* coral skeletons. Quat. Geochronol..

[30] Demarchi B, Collins MJ, Tomiak PJ, Davies BJ, Penkman KEH (2013). Intra-crystalline protein diagenesis (IcPD) in *Patella vulgata*. Part II: Breakdown and temperature sensitivity. Quat. Geochronol..

[31] Weitschat, W. & Wichard, W. Baltic amber. In *Biodiversity of fossils in amber from the major world deposits* (ed. Penney, D.) 80–115 (Siri Scientific Press Manchester, 2010).

[32] Shi G (2012). Age constraint on Burmese amber based on U–Pb dating of zircons. Cretaceous Res..

[33] Walton D, Curry GB (1991). Amino acids from fossils, facies and fingers. Palaeontology.

[34] Oró J, Skewes HB (1965). Free amino-acids on human fingers: the question of contamination in microanalysis. Nature.

[35] Chu PG, Weiss LM (2002). Keratin expression in human tissues and neoplasms. Histopathology.

[36] Vallentyne JR (1964). Biogeochemistry of organic matter—II Thermal reaction kinetics and transformation products of amino compounds. Geochim. Cosmochim. Acta.

[37] Vallentyne JR (1969). Pyrolysis of amino acids in Pleistocene *Mercenaria* shells. Geochim. Cosmochim. Acta.

[38] McCoy VE, Soriano C, Gabbott SE (2018). A review of preservational variation of fossil inclusions in amber of different chemical groups. Earth Environ. Sci. Trans. R. Soc. Edinb..

[39] McCoy VE (2018). Unlocking preservation bias in the amber insect fossil record through experimental decay. PLoS One.

[40] Ross, A., Mellish, C., York, P. & Crighton, B. Burmese amber. In *Biodiversity of fossils in amber from the major world deposits* (ed. Penney, D.) 208–235 (Siri Scientific Press Manchester, 2010).

[41] Lambert JB, Santiago-Blay JA, Wu Y, Levy AJ (2015). Examination of amber and related materials by NMR spectroscopy. Magn. Reson. Chem..

[42] Poinar G, Lambert JB, Wu Y (2007). Araucarian source of fossiliferous Burmese Amber: Spectroscopic and anatomical evidence. J. Bot. Res. Inst. Tex..

[43] Dolezych M, Fischer T, Gröschke A (2011). *Pinuxylon succiniferum* (Goeppert) Kraeusel emend. Dolezych–amberized wood from Goeppert’s type material restudied. Mauritiana (Altenburg).

[44] Alonso J (2000). A new fossil resin with biological inclusions in Lower Cretaceous deposits from Álava (Northern Spain, Basque-Cantabrian Basin). Journal of Paleontology.

[45] Wolfe AP (2009). A new proposal concerning the botanical origin of Baltic amber. Proc. Biol. Sci..

[46] Peñalver, E. & Delclòs, X. Spanish amber. In *Biodiversity of fossils in amber from the major world deposits* (ed. Penney, D.) 236–270 (Siri Scientific Press Manchester, 2010).

[47] Peñalver E, Delclòs X, Soriano C (2007). A new rich amber outcrop with palaeobiological inclusions in the Lower Cretaceous of Spain. Cretaceous Res..

[48] King TE (2014). Identification of the remains of King Richard III. Nat. Commun..

[49] Armstrong DW, Kullman JP, Chen X, Rowe M (2001). Composition and chirality of amino acids in aerosol/dust from laboratory and residential enclosures. Chirality.

[50] Hadorn B, Hanimann F, Anders P, Curtius HC, Halverson R (1967). Free amino-acids in human sweat from different parts of the body. Nature.

[51] Liappis N, Kelderbacher SD, Kesseler K, Bantzer P (1979). Quantitative study of free amino acids in human eccrine sweat excreted from the forearms of healthy trained and untrained men during exercise. Eur. J. Appl. Physiol. Occup. Physiol..

[52] Fritz, P. Chemical studies into the amino acids present in latent fingermarks. (Curtin University, 2015).

[53] Kaufman DS, Manley WF (1998). A new procedure for determining DL amino acid ratios in fossils using reverse phase liquid chromatography. Quat. Sci. Rev..

[54] Hill RL (1965). Hydrolysis of proteins. Adv. Protein Chem..

