# Supplementary material for: Ancient amino acids from fossil feathers in amber
Source: Sci Rep. 2019 Apr 23;9:6420. doi: 10.1038/s41598-019-42938-9 (PMC6478714; doi:10.1038/s41598-019-42938-9)
Supplement: Supplementary file 1 — Supplementary Information for Ancient amino acids from fossil feathers in amber [file 41598_2019_42938_MOESM1_ESM.pdf]

Supplementary Information for

Ancient amino acids from fossil feathers in amber

Victoria E. McCoy, Sarah E. Gabbott, Kirsty Penkman, Matthew J Collins, Samantha Presslee, John Holt, Harrison Grossman, Bo Wang, Monica M. Solórzano Kraemer, Xavier Delclòs, and Enrique Peñalver

This PDF file includes:

Figs. S1
Table S1
Captions for additional data tables S2-S5 (separate files)

Supplemental figures

Supplemental figure S1: Amino acid analyses, including amino acid composition and D/L, of thermal degradation experiments including modern feathers in resin, modern feathers in air, and modern feathers in water. Amino acid abbreviations as in Figure 2.

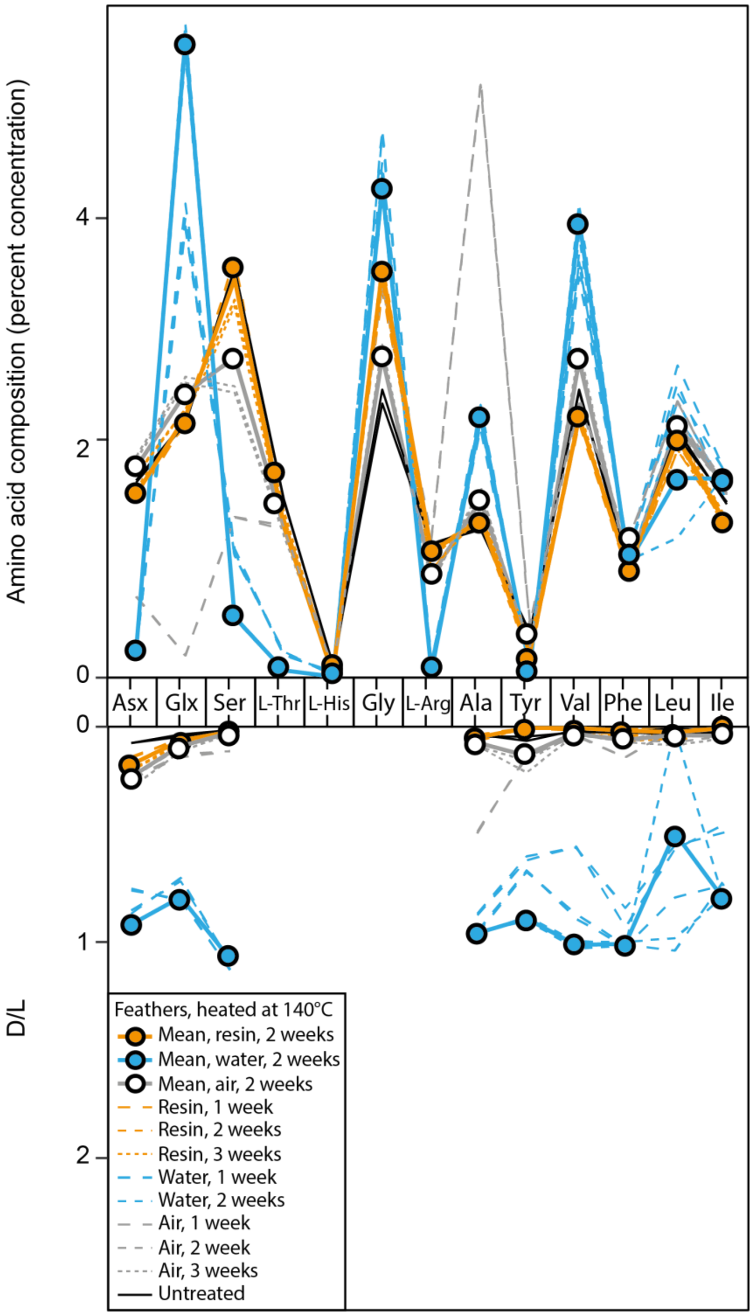

Supplemental table

Supplemental table S1: List of samples

Sample designation	Museum
Baltic amber (44 Ma)	
SMF Be 370	Senckenberg Research Institute and Natural History Museum, Frankfurt
Burmese amber (99 Ma)	
specimen 1*	Nanjing Institute of Geology and Palaeontology, Chinese Academy of Sciences, China
specimen 2*	
specimen 3*	
specimen 4*	
specimen 5*	
specimen 6*	
Spanish amber (105 Ma)	
CES 426	The laboratory of the El Soplao Cave, Celis, Cantabria (Spain) encompassing the Institutional Collection from the El Soplao outcrop
CES 457	

*All Burmese amber specimens were accessioned into the Nanjing Institute of Geology and Paleontology, but these specimens were not given numbers because this institution only assigns specimen numbers for holotypes and paratypes.

Additional data table S2 (separate file): Concentrations of amino acids in successful fossil analyses and experiments with modern feathers in resin

Additional data table S3 (separate file): D/L for each amino acid in successful fossil analyses and experiments with modern feathers in resin

Additional data table S4 (separate file): D/L for amino acids in all fossil samples and control samples, including unsuccessful analyses

Additional data table S5 (separate file): Concentrations for amino acids in all fossil samples and control experiments, including unsuccessful analyses
